# Ghost Ileostomy Versus Loop Ileostomy Following Oncologic Resection for Rectal Cancer: A Systematic Review and Meta-Analysis

**DOI:** 10.1177/15533506231169066

**Published:** 2023-04-04

**Authors:** Tyler McKechnie, Jay Lee, Yung Lee, Léa Tessier, Nalin Amin, Aristithes Doumouras, Dennis Hong, Cagla Eskicioglu

**Affiliations:** 1 Division of General Surgery, Department of Surgery, 3710 McMaster University, Hamilton, ON, Canada; 2 Michael G DeGroote School of Medicine, 12362 McMaster University, Hamilton, ON, Canada; 3 Division of General Surgery, Department of Surgery, University of Calgary, Calgary, AB, Canada; 4 Division of General Surgery, Department of Surgery, St. Joseph Healthcare, Hamilton, ON, Canada

**Keywords:** ghost ileostomy, virtual ileostomy, rectal cancer, tumor-specific mesorectal excision, anastomotic leak

## Abstract

**Objective:**

The aim of this study was to compare ghost ileostomy (GI) and loop ileostomy (LI) in patients undergoing oncologic resection for rectal cancer in terms of postoperative morbidity.

**Summary Background Data:**

LIs are often fashioned to protect downstream anastomoses following oncologic resection for low rectal cancer at medium-to-high risk of anastomotic leak. More recently, GIs have been utilized in patients with low-to-medium risk anastomoses to reduce the rate of unnecessary stomas.

**Methods:**

Medline, Embase, and CENTRAL were systematically searched. Studies investigating the use of GI in patients undergoing oncologic resection for rectal cancer were included. The primary outcomes were anastomotic leak and postoperative morbidity. Secondary outcomes included stoma-related complications and length of stay (LOS). Pairwise meta-analyses were performed with inverse variance random effects.

**Results:**

From 242 citations, 14 studies with 946 patients were included. In comparative studies, 359 patients were undergoing GI and 266 patients were undergoing LI. Pairwise meta-analysis revealed no differences in the prevalence of anastomotic leak (OR 1.40, 95%CI .73-2.68, *P* = .31), morbidity (OR .76, 95%CI .44-1.30, *P* = .32), or LOS (SMD -.05, 95%CI -.33-.23, *P* = .72). International Study Group of Rectal Cancer anastomotic leak grades were as follows: Grade A (GI 0% vs LI 13.3%), Grade B (GI 80.9% vs LI 86.7%), Grade C (GI 19.1% vs LI 0%).

**Conclusions:**

GI appears to be a safe alternative to LI following oncologic resection for rectal cancer. Larger, prospective comparative studies are warranted to evaluate the use of GI in patients deemed to be at low-to-medium risk of anastomotic leak.

## Introduction

Radical oncologic resection remains the mainstay of definitive treatment for most rectal cancers.^1^ Provided there is not tumour involvement of the anal sphincter complex and achieving an appropriate distal margin is feasible, low anterior resection (LAR) with tumor-specific mesorectal excision (TME) or trans-anal total mesorectal excision (TaTME) are the most common surgical approaches. Anastomotic leak following these operations is 1 of the most relevant postoperative complications, with incidence rates of 10-15%.^2–4^ Following mid-to-low colorectal or coloanal anastomoses, de-functioning loop ileostomies (LIs) are commonly constructed to protect the downstream anastomosis and attenuate the consequences of a potential anastomotic leak. Systematic reviews have demonstrated the benefits of this approach in select patients, such as those with low anastomoses, receiving immunosuppressants, and obese males.^5,6^ However, there can be significant morbidity associated with loop ileostomy, including complications such as high-output ileostomy and parastomal hernia.^7,8^ Overall morbidity can range from 30 to 65% in these patients, and readmissions can occur in up to 40% of patients following formation of an LI.^9,10^ Furthermore, stomas may have significant impact on patient quality-of-life (QoL).^11,12^ Lastly, the patient is subjected to another operation for ileostomy reversal, which comes with its associated anesthetic risks, hospital stay, and postoperative morbidity.^10,13^

As such, there has been the recent development, study, and implementation of ghost or virtual ileostomies.^14,15^ Ghost ileostomy (GI) formation involves temporarily securing a loop of distal ileum to the intraperitoneal anterior abdominal wall with the use of a vessel loop, suture, or drain passing underneath the bowel and through the associated mesentery.^16,17^ Some authors describe the creation of a fascial defect at the time of GI formation, which has been termed a “parietal split.”^18^ Should signs of anastomotic leak arise, the GI can be converted to a LI in the operating room or even under local anesthetic at the bedside. If there are no signs of anastomotic leak in the early postoperative period, the vessel loop can be removed, releasing the underlying loop of small bowel, and thus obviating the need for a LI and formal LI reversal.

The first description of this technique was by Sacchi et al in 2007.^14^ Subsequently, several smaller observational cohort studies have been published demonstrating the advantages of GI following TME.^15,18-20^ 1 randomized controlled trial (RCT) was conducted in 2015, which compared the use of GI against no stoma formation.^16^ The authors found that in patients deemed to be at medium-risk of anastomotic leak preoperatively, only 3 patients (5.5%) experienced anastomotic leak requiring conversion of the ghost ileostomy to a diverting ileostomy at the bedside under local anesthesia. A recent systematic review qualitatively described the operative technique for GIs, but did not quantitatively synthesis outcome data, nor did it compare GI to conventional LI.^21^ To date, there is no meta-analysis evaluating the use of GI following TME for rectal cancer. Therefore, we sought out to perform this systematic review and meta-analysis to summarize the safety profile and outcomes of this novel technique, and compare it in terms of anastomotic leak and postoperative morbidity to LI.

## Methods

### Search Strategy

The following databases covering the period from database inception through July 2022 were searched: Medline, EMBASE, and Cochrane Central Register of Controlled Trials (CENTRAL). The search was designed and conducted by a medical research librarian with input from study investigators. Search terms included “rectal neoplasms”, “virtual ileostomy”, “ghost ileostomy”, “loop ileostomy”, and more (complete search strategy available in Supplement Appendix 1). The references of studies meeting inclusion criteria were searched manually to ensure that all relevant articles were included. This systematic review and meta-analysis is reported in accordance with the Preferred Reporting items for Systematic Reviews and Meta-Analyses (PRISMA) and the Meta-Analysis of Observational Studies in Epidemiology (MOOSE).^22,23^ The study protocol was registered on the International Prospective Register of Systematic Reviews (PROSPERO) a priori (CRD42021271699). Local ethics review board approval was not required for this study.

### Study Selection

Articles were eligible for inclusion if they were randomized controlled trials (RCTs), cohort studies, case-control studies, cross-sectional studies, case series’, or letters reporting primary data comparing GI formation to LI formation in the context of TME for rectal cancer and reported prevalence of anastomotic leak and/or 30-day postoperative morbidity. Relevant single-arm studies evaluating patients receiving GIs following TME for rectal cancer were also included. Studies with patients undergoing rectal resection for non-rectal cancer pathologies were excluded. Studies were not discriminated on the basis of language. Conference abstracts were considered for inclusion. Lastly, case reports, systematic reviews, meta-analyses, and editorials were excluded.

### Outcomes Assessed

The primary outcomes were anastomotic leak rate and 30-day overall postoperative morbidity. Anastomotic leak was defined on the basis of clinical and/or radiographic findings in the included studies. Clinical definitions included, purulent or feculent contents in an intraperitoneal drain or feculent drainage from a surgical incision.^19^ Radiographic definitions included extravasation of water-soluble intra-luminal contrast on computed tomography (CT) or a pelvic abscess adjacent to the anastomosis on CT.^17,19^ Postoperative morbidity was defined as any documented deviation from the expected postoperative course documented in patient medical records or database records.

Secondary outcomes included: (1) operative time in minutes; (2) postoperative length of stay (LOS) in days; (3) reoperation; (4) readmission and/or re-presentation to the emergency department; and (5) 30-day overall postoperative mortality. Postoperative LOS was defined as the time from the end of the index procedure to the time the patient left the hospital following their index procedure in all included studies. SSIs were defined according to the Centre for Disease Control and Prevention.^24^

### Data Extraction

Two reviewers independently evaluated the systematically searched titles and abstracts using a standardized, pilot-tested form. Discrepancies that occurred at the title and abstract screening phases were resolved by inclusion of the study. At the full-text screening stage, discrepancies were resolved by consensus between the 2 reviewers. If disagreement persisted, a third reviewer was consulted. 2 reviewers independently conducted data extraction into a data collection form designed a priori. The extracted data included study characteristics (eg, author, year of publication, study design), patient demographics (eg, age, gender, body mass index [BMI], comorbidities), treatment characteristics (eg, operative approach, index operation, neoadjuvant therapy, operative time), postoperative morbidity (eg, anastomotic leak, SSI, readmission, reoperation), and LOS.

### Risk of Bias Assessment and Certainty of Evidence

Risk of bias for observational studies was assessed using the Risk of Bias in Non-randomized Studies – of Interventions (ROBINS-I) assessment tool.^25^ Risk of bias for RCTs was assessed using the Cochrane Risk of Bias Tool for Randomized Controlled Trials 2.0.^26^ Quality of evidence for estimates derived from meta-analyses were assessed by Grading of Recommendations, Assessment, Development and Evaluation (GRADE).^27^ Two reviewers assessed the risk of bias and certainty of evidence independently. Discrepancies were discussed amongst the reviewers until consensus was reached.

### Statistical Analysis

All statistical analyses and meta-analyses were performed on STATA version 14 (StataCorp, College, TX) and Cochrane Review Manager 5.3 (London, United Kingdom). The calculations and organization of results into a summary of findings table was done using the GRADEPro software.^28^ The threshold for statistical significance was set a priori at a *P* of <.05. A pairwise meta-analysis was performed using an inverse variance, random effects model for all meta-analyzed outcomes. Pooled effect estimates were obtained by calculating the mean difference (MD) in outcomes for continuous variables and odds ratios (OR) for dichotomous variables along with their respective 95% confidence intervals (CI) to confirm the effect size estimation. In addition, mean and standard deviation (SD) was estimated for studies that only reported median and interquartile range using the method described by Wan et al.^29^ For studies that did not report standard deviation or interquartile range, we contacted the authors for missing data. Data was presumed to be unreported if no response was received from study authors within 2 weeks from the index point of contact. Missing SD data were then calculated according to the prognostic method.^30^ Assessment of heterogeneity was completed using the inconsistency (I^2^) statistic. An I^2^ greater than 50% was considered to represent considerable heterogeneity.^31^ Bias in meta-analyzed outcomes was assessed with funnel plots when data from more than 10 studies were included in the analysis.^32^ A leave-one-out sensitivity analysis was performed by iteratively removing 1 study at a time from the inverse variance, random effects model to ensure that pooled effect estimates were not driven by a single study. Additionally, a sensitivity analysis on the basis of study publication date and high risk of bias according to the Cochrane Risk of Bias Tool for Randomized Controlled Trials 2.0 and ROBINS-I was performed to ensure that pooled effect estimates were not impacted by low quality, potentially biased data. For outcomes that were reported in less than 3 studies, a systematic narrative summary was provided.^33^

## Results

### Study Characteristics

**Figure 1.**
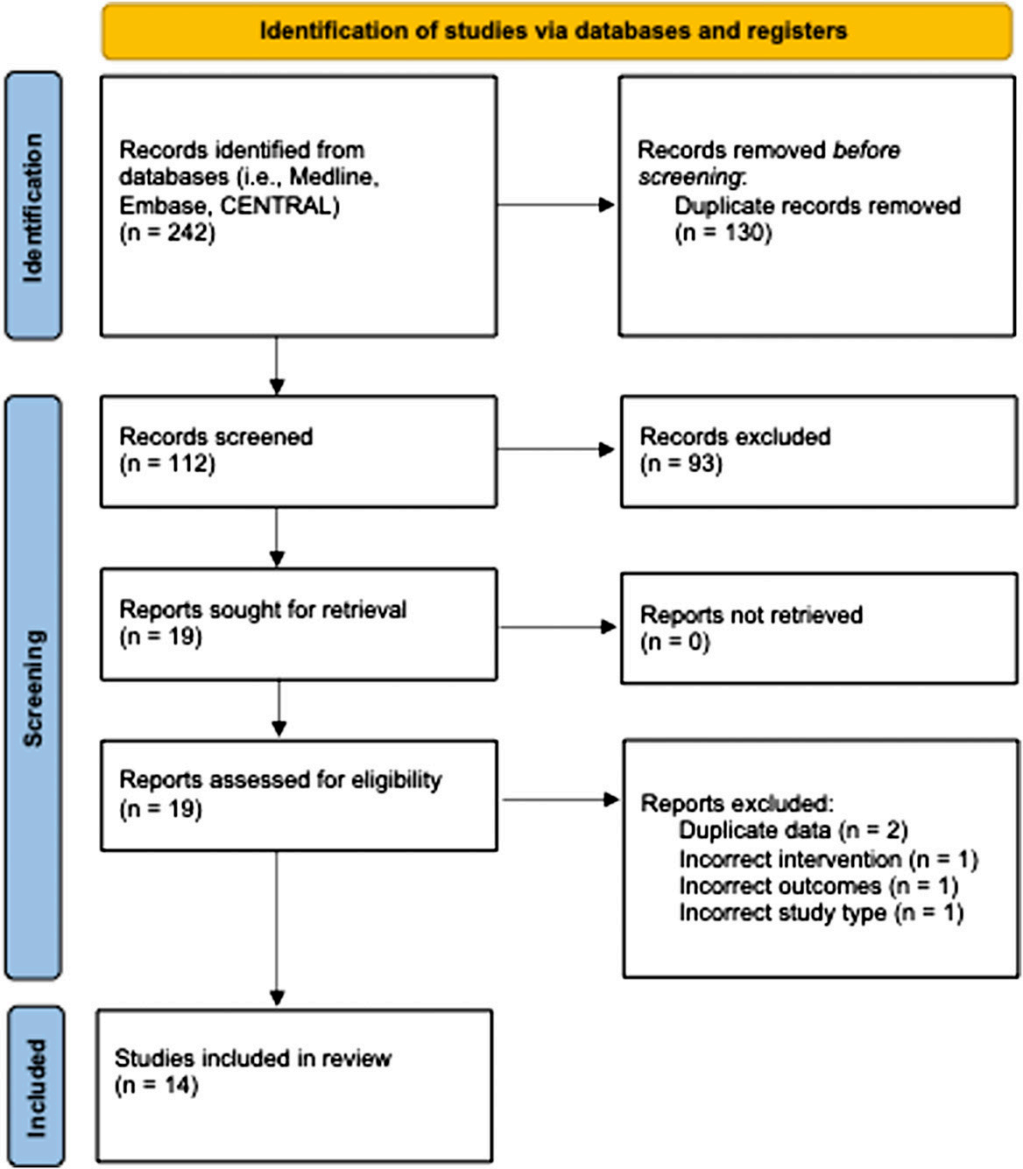
PRISMA diagram – transparent reporting of systematic reviews and meta-analysis flow diagram outlining the search strategy results from initial search to included studies.^22^

From 242 citations, 14 studies (7 retrospective cohorts, 3 prospective cohorts, 2 letters, 1 RCT, and 1 conference proceeding) with 628 patients undergoing GI (47.3% female, mean age: 66.5, mean BMI: 26.8), 266 patients undergoing LI (41.6% female, mean age: 65.0, mean BMI: 25.7) and 52 patients not receiving a stoma (48.1% female, mean age: 69.0, mean BMI: 29.2) were included.^14-20,34-40^ A PRISMA flow diagram of the study selection process is illustrated in Figure 1.^22^ Included studies were conducted between 2007 and 2021. The study periods of the included studies ranged from 1997 to 2019. Detailed study characteristics are reported in Table 1.

**Table 1.** Study characteristics of the included studies.

Author, Year	Arms	N	Mean Age, y (SD)	% Female	Mean BMI (SD)	Mean ASA Class (SD)	AJCC Rectal Cancer Stage
Sacchi, 2007 (retrospective)	GI	107	68 (39-85)^b^	43.9	—	—	—
Micinni, 2010 (retrospective)	GI	36	62.5 (39-88)^a^	44.4	—	—	—
Cerroni, 2011 (prospective)	GI	20	54-86^c^	40.0	—	—	—
Sacchi, 2011 (retrospective)	GI	44	—	—	—	—	—
	LI	35	—	—	—	—	—
Gulla, 2011 (prospective)	GI	18	72 (43-86)^b^	44.4	22.7 (6.5)	1.4 (.6)	II – 12 (66.7)
							III – 6 (33.3)
	LI	27	73 (48-88)^b^	44.4	23.6 (8.2)	1.5 (.6)	II – 20 (74.1)
							III – 7 (25.9)
Mori, 2013 (retrospective)	GI	168	—	—	—	—	—
	LI	17	—	—	—	—	—
Giarratano, 2014 (retrospective)	GI	15	—	—	—	—	—
Marrosu, 2014 (letter)	GI	22	65^b^	73.5	—	—	—
	LI	27	65^b^	73.5	—	—	—
Mari, 2015 (RCT)	GI	55	71.0 (7.6)	52.7	29.3 (2.6)	—	I – 6 (10.9)
							II – 19 (34.5)
							III – 25 (45.5)
							IV – 5 (9.1)
	No stoma	52	69.0 (8.2)	48.1	29.2 (3.0)	—	I – 5 (9.6)
							II – 22 (42.3)
							III – 22 (42.3)
							IV – 3 (5.8)
Jimenez, 2019 (retrospective)	GI	24	—	56.2	—	—	—
Palumbo, 2019 (retrospective)	GI	32	65.7 (10.0)	56.2	24.0 (3.4)	1.8 (.6)	I – 14 (43.8)
							II – 2 (6.5)
							III – 13 (40.6)
							IV – 3 (9.4)
	LI	50	68.8 (11.0)	31.6	25.2 (4.4)	1.8 (.6)	I – 24 (44.0)
							II – 14 (28.0)
							III – 10 (20.0)
							IV – 4 (8.0)
Morales-Conde, 2020 (prospective)	GI	12	61.1 (10.6)	25.0	27.4 (3.6)	1.9 (.7)	I – 5 (41.7)
							II – 7 (58.3)
Zenger, 2021 (retrospective)	GI	42	61.0 (11.0)	42.9	27.4 (4.6)	2.1 (.7)	I – 14 (33.4)
							II – 8 (19.0)
							III – 20 (47.6)
	LI	81	60.0 (11.0)	35.8	26.6 (3.8)	2.1 (.6)	I – 24 (29.6)
							II – 20 (24.7)
							III – 37 (45.7)
Khan, 2021 (letter)	GI	33	—	—	—	—	
	LI	29	—	—	—	—	—

^a^Mean (range).

^b^Median (range).

^c^Range.

RCT, randomized controlled trial; GI, ghost ileostomy; LI, loop ileostomy; y, years; SD, standard deviation; BMI, body mass index; ASA, American Society of Anesthesiologists; AJCC, American Joint Committee on Cancer.

### Operative Technique

All included patients were undergoing sphincter-sparing oncologic resection for rectal cancer. The most common oncologic resections were an anterior resection (AR) or low anterior resection (LAR) (n = 942, 99.6%). Eleven studies reported operative approach. Overall, 39.2% of patients underwent minimally invasive oncologic resection. The mean distance of the anastomosis from the anal verge was 8.3 cm in patients undergoing GI and 7.0 cm in patients undergoing LI. In the retrospective cohort study published by Palumbo et al, patients in the GI group had significantly more proximal anastomoses compared to patients in the LI group (10.4 cm vs 6.7 cm, *P* < .05).^35^ Treatment characteristics of the included studies are reported in Table 2.

**Table 2.** Treatment Characteristics of the Included Studies.

Author, Year	Arms	N	% Lap	N Type of Resection (%)	Mean Height of Anastomosis, cm (SD)	N Neoadjuvant Therapy (%)	Parietal Split (Y/N)	Ghost Ileostomy Technique
Sacchi, 2007 (retrospective)	GI	107	—-	LAR – 107 (100)	—	—	Y	• 1 cm transverse incision in right iliac fossa through all layers of abdominal wall
								• Passage of vessel loop through incision and through a defect created in the ileal mesentery 25-40 cm proximal to the IC valve
								• Approximation of the vessel loop at the level of the skin with silk suture
Micinni, 2010 (retrospective)	GI	36	0	LAR – 36 (100)	—	—	Y	• Small incision made in right iliac fossa through all the layers of the abdominal wall, through which an elastic tape was passed through
								• Elastic tape subsequently passed through a defect created in the mesentery of the distal third of the ileum
								• The elastic tape was secured to a gauze pad at the level of the anterior abdominal wall
Cerroni, 2011 (prospective)	GI	20	0	LAR – 20 (100)	—	9 (45.0)	Y/N	• No parietal split: A prolene stitch was passed through the skin at the level of the right iliac fossa, into the abdomen, underneath a loop of ileum, and then back through the anterior abdominal wall, and the stitch was secured at the level of the anterior abdominal wall with non-absorbable suture
								• Parietal split: An incision was made in the right iliac fossa through all the layers of abdominal wall, a pediatric Robinson catheter was passed through the incision, through a defected created in the mesentery of the ileum, and then back through the abdominal wall incision where it was fixed to the skin with absorbable suture
Sacchi, 2011 (retrospective)	GI	44	17.7	LAR – 75 (94.9)	—	—	—	• Vessel looped passed through a defect created in the terminal ileum mesentery at the time of laparotomy specimen extraction
	LI	35		TaTME – 4 (5.1)	—	—	—	• Vessel loop passed through an incision/trochar in the right iliac fossa and secured at the level of the skin with suture
Gulla, 2011 (prospective)	GI	18	0	AR – 18 (100)	6.1 (3.5-8.0)^a^	18 (100)	N	• Vessel loop passed through a defect created in the mesentery of the ileum 40 cm proximal to the IC valve
								• Vessel loop exteriorized through the right iliac fossa via the BERCI fascial closer
	LI	27	0	AR – 27 (100)	6.3 (3.5-8.0)^a^	27 (100)	—	• Vessel loop ends attached with a surgical clip extra-corporeally and then sutured at the level of the skin
Mori, 2013 (retrospective)	GI	168	17.8	LAR – 168 (100)	—	15 (8.9)	Y	—
	LI	17		LAR – 17 (100)	—	—	—	
Giarratano, 2014 (retrospective)	GI	15	0	AR – 15 (100)	—	—	Y	• Small incision made in right iliac fossa through all the layers of the abdominal wall, through which a vessel loop was passed
								• Vessel loop passed through a defect created in the mesentery of the ileum 10 cm proximal to the IC valve
								• Vessel loop passed back through the incision and secured at the level of the skin with a stoma rod
								• Silk stitch marked the afferent loop
Marrosu, 2014 (letter)	GI	22	0	AR – 22 (100)	—	—	N	• 1-2 mm incision made in right iliac fossa through all the layers of the abdominal wall, through which a vessel loop was passed
	LI	27	0	AR – 27 (100)	—	—	—	• Vessel loop passed through a defect created in the mesentery of the ileum 20 cm proximal to the IC valve
								• Vessel loop passed back through the incision and secured at the level of the skin with suture
								• Silk stitch marked the afferent loop
Mari, 2015 (RCT)	GI	55	100	AR – 55 (100)	—	37 (67.3)	Y	• A vessel loop was passed through a defect created in the terminal ileum mesentery laparoscopically
	No stoma	52	100	AR – 52 (100)	—	32 (61.5)	—	• Vessel loop exteriorized through a 10 mm port in the right iliac fossa
								• Vessel loop secured at the level of the skin
Jimenez, 2019 (retrospective)	GI	24	—	LAR – 24 (100)	—	—	—	—
Palumbo, 2019 (retrospective)	GI	32	36.6	LAR – 32 (100)	10.4 (3.4)	13 (40.6)	Y	• Identical to technique described by Micinni et al (2010)
	LI	50		LAR – 50 (100)	6.7 (2.8)	24 (48.0)	—	
Morales-Conde, 2020 (prospective)	GI	12	100	AR – 12 (100)	8.2 (3.1)	5 (41.7)	Y	• A vessel loop was passed through a defect created in the terminal ileum mesentery 30-40 cm proximal to the IC valve laparoscopically
								• Vessel loop exteriorized through a 10 mm port in the right iliac fossa
								• Vessel loop secured at the level of the skin
								• Surgical clip marked the afferent loop
Zenger, 2021 (retrospective)	GI	42	83.3	LAR – 42 (100)	7.8 (1.4)	10 (23.8)	N	• Small incision was made in the right iliac fossa through which a vessel loop was passed
	LI	81	79.0	LAR – 81 (100)	7.4 (1.5)	46 (56.8)	—	• Vessel loop passed through a defect created in the ileal mesentery 20-cm proximal to the IC valve
								• Approximation of the vessel loop at the level of the skin with silk suture
Khan, 2021 (letter)	GI	33	—	LAR – 33 (100)	—	—	N	—
	LI	29	—	LAR – 29 (100)	—	—	—	

^a^Median (range).

N, number of patients; GI, ghost ileostomy; LI, loop ileostomy; SD, standard deviation; Y, yes; N, no; Lap, laparoscopic surgery; LAR, low anterior resection; cm, centimeters; mm, millimeter; TaTME, transanal total mesorectal excision; AR, anterior resection; IC, ileo-cecal.

There was significant heterogeneity in the techniques reported for fashioning the GI. The majority of included studies (8/12, 66.7%) employed a parietal splitting technique (ie, creating a large enough fascial defect that would allow for fashioning of a loop ileostomy without re-incising it at the time of re-operation).^14-16,18,20,35,37,39^ In the studies reporting a no-parietal splitting technique, fascial incisions were reported as 1-2 mm in size, whereas in studies reporting a parietal splitting technique, fascial incisions were 10 mm or greater. The most commonly used material for approximating the loop of ileum to the anterior abdominal wall were vessel loops (8/11, 72.7%), followed by elastic tape (2/11, 18.2%), prolene stitch (1/11, 9.1%), and pediatric Robinson catheters (1/11, 9.1%). Of the 6 studies that reported distance of GI formation from the ileal-cecal valve, all were within 40 cm.^14,17,19,36,37,39^ If no signs of anastomotic leak were observed postoperatively, the GI was released between postoperative days 8 and 15 in all 5 studies that reported these data specifically.^17-20,35^ Specific operative details for each of the included studies are reported in Table 2.

### Anastomotic Leak

**Figure 2.**
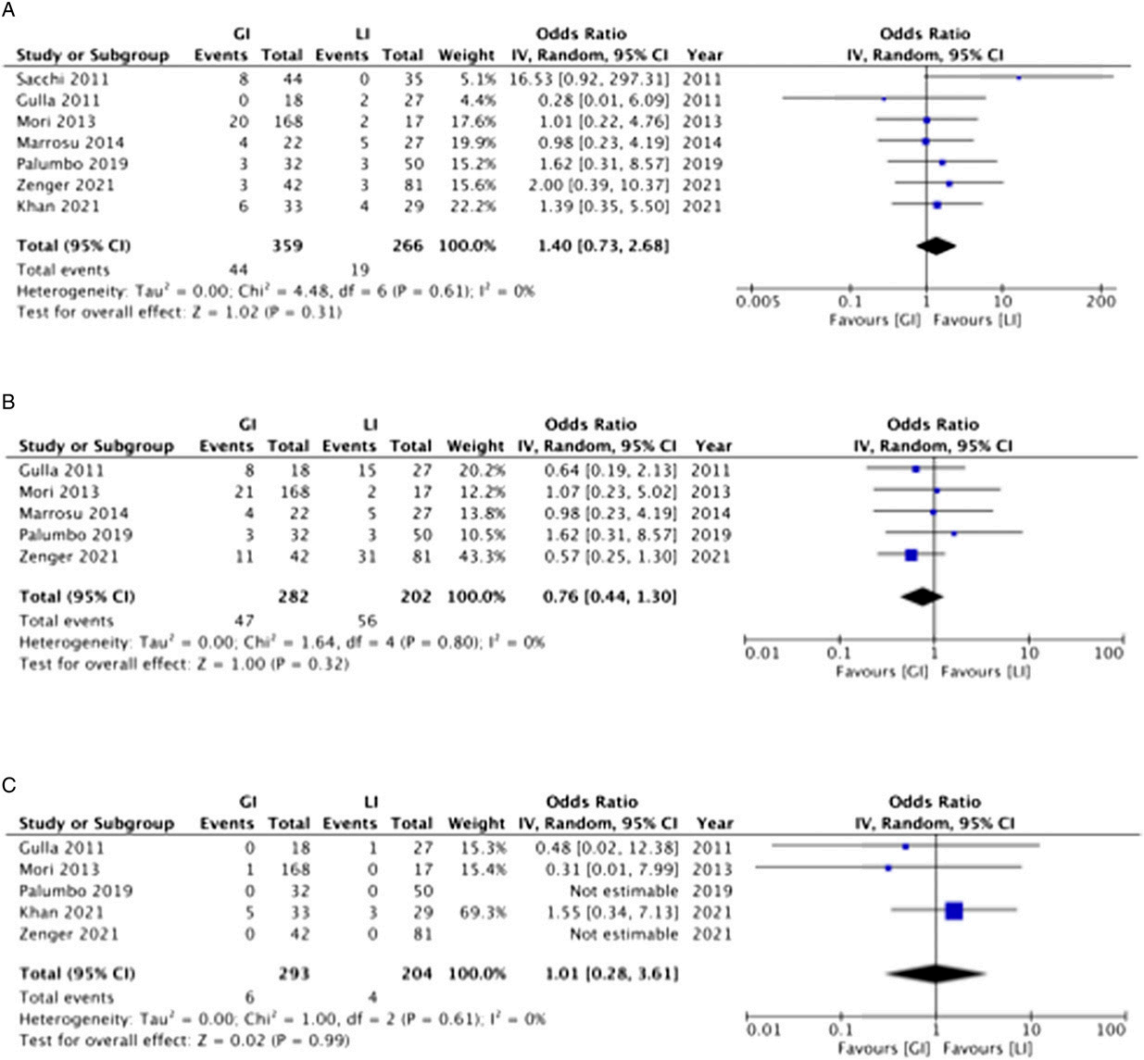
Anastomotic leak (A), 30-day postoperative morbidity (B), and 30-Day postoperative mortality – random effect inverse variance meta-analysis comparing ghost ileostomy and loop ileostomy.

Seven included studies compared GI and LI in terms of anastomotic leak.^17,19,20,34-36,38^ There was no significant difference between groups in the proportion of patients experiencing anastomotic leak (GI: 12.3% vs LI: 7.1%, OR 1.40, 95%CI .73-2.68, *P* = .31, I^2^ = 0%) (Figure 2(A)). In the only RCT included, that compared GI to no stoma, the incidences of anastomotic leak were 5.4% (3/55) and 7.7% (4/52) in the GI and no stoma groups, respectively.^16^ Thirteen of the included studies graded anastomotic leaks according to the International Study Group of Rectal Cancer (ISGRC).^14-20,35-40^ In the GI group, anastomotic leak grades were as follows: Grade A 0%, Grade B 80.9%, and Grade C 19.1%. In the LI group, anastomotic leak grades were as follows: Grade A 13.3%, Grade B 86.7%, and Grade C 0%. Grade A leaks were more common in the LI group (OR .02, 95%CI .00-.40, *P* = .01, I^2^ = 0%). There was no significant difference in incidence of Grade B (OR 1.27, 95%CI .37-4.37, *P* = .71, I^2^ = 0%) and Grade C leaks (OR 3.28, 95%CI .49-21.86, *P* = .22, I^2^ = 12%) between patients undergoing GI and LI.

### Postoperative Morbidity and Mortality

There was no significant difference between groups in the proportion of patients experiencing 30-day postoperative morbidity (5 studies; GI: 16.7% vs LI: 27.7%, OR .76, 95%CI .44-1.30, *P* = .32, I^2^ = 0%) (Figure 2(B)).^17,19,20,35,36^ There was no significant difference between groups in the proportion of patients experiencing 30-day postoperative mortality (5 studies; GI: 2.0% vs LI: 2.0%, OR .1.01, 95%CI .28-3.61, *P* = .99, I^2^ = 0%) (Figure 2(C)).^17,19,20,34,35^

Three of the included comparative studies compared GI and LI in terms of stoma-related morbidity (eg, high-output ileostomy, stoma prolapse, stoma retraction, etc.). Gulla et al found a significantly greater proportion of patients in the LI group experiencing stoma-related morbidity (GI: 2/18 (7.4%) vs LI: 13/27 (48.1%), *P* = .04).^17^ Similarly, Palumbo et al demonstrated a stoma-related morbidity incidence of 10% (5/50) in the LI group, compared to no patients in the GI group.^35^ There was no stoma-related morbidity reported in the retrospective cohort study published by Mori et al in 2013.^20^ A meta-analysis was not performed given the heterogeneity in reported complications. Postoperative morbidity for each of the included studies is reported in Table 3.

**Table 3.** Postoperative Outcomes Reported in the Included Studies.

Author, Year	Arms	N	Mean LOS, d (SD)	N Mortality (%)	N Morbidity (%)	N Stoma-Related Complications (%)	N Anastomotic Leak (%)	ISGRC Leak Grade	N GI Converted to LI (%)
Sacchi, 2007 (retrospective)	GI	107	—	—	14.1 (13.1)	—	14 (13.1)	B – 14 (100)	Local – 14(100)
Micinni, 2010 (retrospective)	GI	36	—	0	4 (11.1)	0	4 (11.1)	B – 4 (100)	Local – 4 (100)
Cerroni, 2011 (prospective)	GI	20	—	1 (5.0)	5 (25.0)	0	1 (5.0)	C – 1 (100)	OR – 1 (100)
Sacchi, 2011 (retrospective)	GI	44	—	—	—	—	8 (18.2)	B – 8 (100)	Local – 8 (100)
	LI	35	—	—	—	—	0	—	—
Gulla, 2011 (prospective)	GI	18	8.8 (2.1)	0	8 (44.4)	2 (7.1)	0	—	—
	LI	27	8.6 (2.1)	1 (3.7)	15 (55.5)	13 (48.1)	2 (7.4)	B – 2 (100)	—
Mori, 2013 (retrospective)	GI	168	—	1 (.6)	21 (12.5)	0	20 (11.9)	B – 18 (90.0)	Local – 13 (72.2)
								C – 2 (10.0)	OR – 5 (27.8)^a^
	LI	17	—	0	2 (11.8)	0	2 (11.8)	A – 2 (100)	—
Giarratano, 2014 (retrospective)	GI	15	—	0	3 (20.0)	—	3 (20.0)	B – 2 (66.7)	Local – 2 (66.7)
								C – 1 (33.3)	OR – 1 (33.3)
Marrosu, 2014 (letter)	GI	22	13.6 (3.0)	—	4 (18.2)	—	4 (18.2)	C – 4 (100)	—
	LI	27	13.6 (3.0)	—	5 (18.5)	—	5 (18.5)	B – 5 (100)	—
Mari, 2015 (RCT)	GI	55	6.3 (1.6)	—	3 (5.4)	0	3 (5.4)	B – 3 (100)	Local – 3 (100)
	Nostoma	52	6.3 (1.7)	—	4 (7.7)	0	4 (7.7)	C – 4 (100)	—
Jimenez, 2019 (retrospective)	GI	24	—	—	4 (16.7)	—	4 (16.7)	C – 4 (100)	OR – 4 (100)
Palumbo, 2019 (retrospective)	GI	32	—	0	3 (9.4)	—	3 (9.4)	B – 3 (100)	Local – 3 (100)
	LI	50	—	0	3 (5.8)	4 (8.0)	3 (5.8)	B – 3 (100)	—
Morales-Conde, 2020 (prospective)	GI	12	10.9 (10.0)	0	1 (8.3)	—	1 (8.3)	B – 1 (100)	—
Zenger, 2021 (retrospective)	GI	42	7.8 (2.2)	0	11 (26.2)	—	3 (7.1)	B – 2 (66.7)	Local – 2 (66.7)
								C – 1 (33.3)	OR – 1 (33.3)
	LI	81	8.2 (3.5)	0	31 (38.3)	—	3 (3.7)	B – 3 (100)	—
Khan, 2021 (letter)	GI	33	—	5 (15.2)	—	—	6 (18.2)	—	6 (100)^b^
	LI	29	—	3 (10.3)	—	—	4 (13.8)	—	—

^a^Two of these patients required formation of a permanent end colostomy at the time of take back to the operating room.

^b^Location of re-intervention not specified.

N, number of patients; GI, ghost ileostomy; LI, loop ileostomy; SD, standard deviation; LOS, length of stay, ISGRC, International Study Group of Rectal Cancer; d, days; Local, under local anesthesia at the bedside; OR, operating room.

### Length of Stay

There was no significant difference between groups in terms of postoperative LOS (3 studies; SMD −.05, 95%CI −.33 to .23, *P* = .72, I^2^ = 0%) (Supplemental Figure 1).^17,19,36^ Two of the included comparative studies reported the proportion of patients requiring readmission to hospital within 30-days of their index procedure. Zenger et al found a significant increase in the prevalence of readmission in patients undergoing LI compared to GI (22.7% vs 4.7%, *P* = .01).^19^ Palumbo et al reported 1 readmission (2.0%) in the LI group and none in the GI group.^35^ The single-armed retrospective cohort study by Micinni et al reported no readmissions in patients undergoing GI creation (0/36).^15^

### Risk of Bias

**Figure 3.**
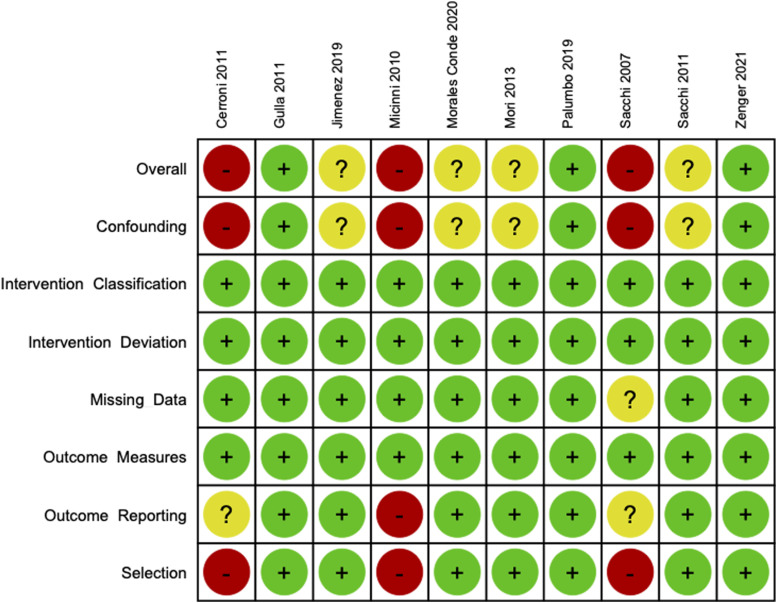
Risk of Bias in Non-randomized Studies of Interventions (ROBINS-I) assessment tool results per individual observational study.

Figure 3 presents the risk of bias analyses according to the ROBINS-I for the included observational studies. The pooled risk of bias analysis according to the ROBINS-I for included observational studies is presented as Supplementary Figure 2. Overall, 3 studies were deemed to be at low risk of bias, 4 studies were deemed to be at unclear risk of bias, and 3 studies were deemed to be at high risk of bias. All 3 studies at high risk of bias had high risk of residual confounding and did not control for selection bias.^14,15,18^ The 3 studies at high risk of bias were also the oldest included studies and single armed studies not included in meta-analyses. All included observational studies were found to be at low risk of bias from classification of the intervention, deviation from the intervention, missing data, and outcome measures.

The risk of bias analysis according to the Cochrane Risk of Bias Tool for Randomized Controlled Trials 2.0 for the only included RCT (Mari et al) found the study to have some concern for bias.^16^ The risk of bias as a result of participant allocation and outcome reporting were unclear, while all other domains (ie, randomization, adherence, missing data, outcome measures) were deemed to present a low risk of bias.

Risk of bias analysis was not performed for Giarratano et al, Marrosu et al, or Khan et al due to study design (ie, conference abstract, letter).^34,36,39^

### Certainty of Evidence

**Figure 4.**
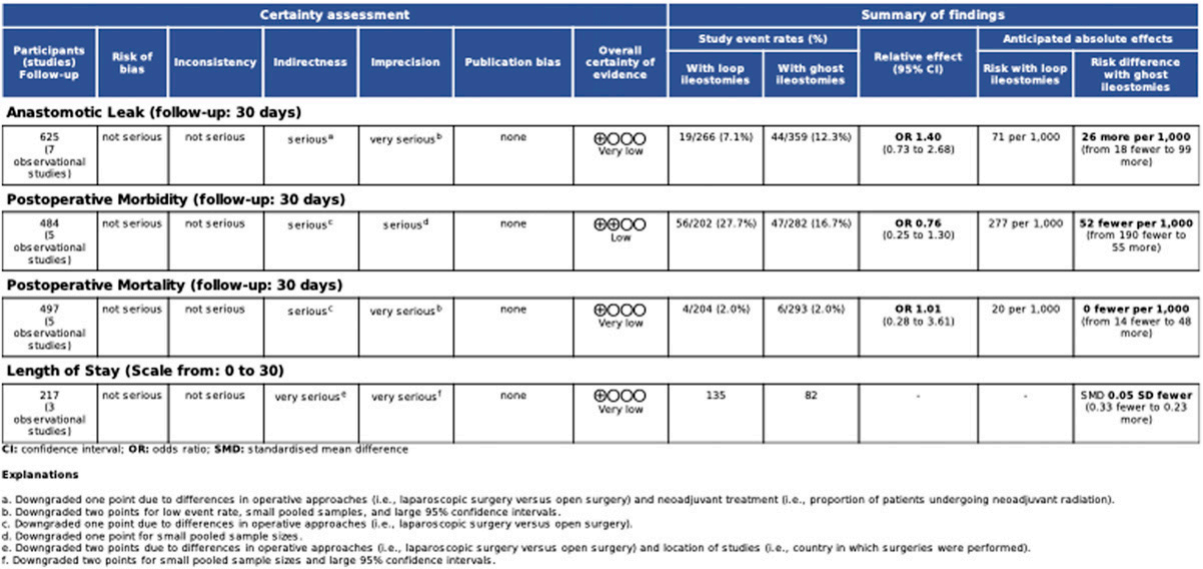
GRADE certainty of evidence summary table for meta-analyses.

The GRADE certainty of evidence summary table is presented in Figure 4. Overall certainty of evidence of 30-day postoperative morbidity was low. For the remaining meta-analyzed outcomes (ie, anastomotic leak, postoperative mortality, LOS), overall certainty of evidence was very low. All outcomes were downgraded due to indirectness and imprecision. Variability in operative approaches for the index operations (ie, laparoscopic, open), proportion of patients undergoing neoadjuvant radiotherapy, and geographic location of study all contributed to serious or very serious concern for indirectness. Small pooled sample sizes, low event rates, and wide 95% confidence intervals lead to serious or very serious concern for imprecision. There were no major concerns with risk of bias or inconsistency across all studies.

## Discussion

GI formation in patients with low-to-medium risk colorectal anastomoses was first described in 2007 and has since been studied extensively in small, observational cohort studies. This was the first systematic review and meta-analysis to quantitatively analyze the efficacy and safety of GIs compared to LIs in this patient population. Low to very low certainty evidence demonstrated no significant difference in the risk of anastomotic leak between GI and LI (GI: 12.3% vs LI: 7.1%, OR 1.40, 95%CI .73-2.68, *P* = .31), nor was there an observed difference in postoperative morbidity (OR .76, 95%CI .44-1.30, *P* = .32) or mortality (OR .1.01, 95%CI .28-3.61, *P* = .99). LOS was similar between the 2 groups (SMD −.05, 95%CI −.33 to .23, *P* = .72).

Narrative review in the present study demonstrated significantly more stoma-associated morbidity in the LI group. Stoma-associated morbidity can include high-output ileostomies, stoma retraction, stoma stenosis, stoma prolapse, parastomal hernia, and more.^41^ High-output ileostomies in particular can occur in 5-25% of cases following TME for rectal cancer with diverting loop ileostomy and may account for up to a 17% readmission rate following these procedures.^42-45^ Readmissions for these patients can cost upwards of $4000 per patient.^46^ In addition, ileostomies can have a detrimental impact on patient quality of life (QoL).^47,48^ Following TME with LI formation, patients tend to have significantly worse QoL than patients who undergo a similar oncologic resection without LI formation (eg, anterior resection).^47^ QoL is then significantly improved following LI reversal.^48^ Only 1 of the studies included in the present review evaluated QoL data. Gullà et al demonstrated a significant improvement in 3-month QoL on the Stoma Quality of Life Index in GI patients.^17^ Future studies comparing GI and LI should aim to incorporate QoL analyses to determine whether a significant benefit exists favoring the GI group. Altogether, LI creation is not a benign procedure with significant morbidity, associated healthcare cost, and detrimental effects on patient QoL and thus reducing the number of patients with low-to-medium risk colorectal anastomoses receiving an LI through formation of a GI can have significant impact.

Ultimately, the safe application of GI is predicated on accurately predicting the risk of anastomotic leak. Patient, treatment, and intraoperative factors must be carefully considered in each individual case prior to proceeding with fashioning of a GI. Patient factors such as older age, male sex, high American Association of Anesthesiologists (ASA) score, malnutrition, and chronic steroid use increase the risk.^49,50^ Thus, the presence of several of these should likely influence the clinician to proceed with LI formation.^49,50^ Similarly, neoadjuvant radiation and anastomoses within 5-6 cm of the anal verge should prompt strong consideration for fashioning an LI.^49,50^ There are predictive scoring systems such as the Colon Leakage Score (CLS) and the Calcium Score for these patients.^51,52^ Similarly, the PROCOLE prognostic index utilizes a risk factors approach to the development of an index, which they have since developed into a free software that physicians can use at the time of surgery.^53^ Application of these scores as well as novel intraoperative techniques, such as near infrared fluorescence angiography with indocyanine green or intraoperative air leak tests, should inform the anastomotic leak risk for each individual patient.^54-56^ The benefit of avoiding an LI and the associated morbidity and the risk of having an uncontrolled anastomotic leak offered by GI formation are likely most optimally balanced in patients at low-to-medium risk of leak.

While the risk of anastomotic leak may not be significantly increased in these patients undergoing GI formation as opposed to LI formation, the consequences of the leak may differ. In the present study, the leaks experienced by the LI group were significantly more likely to be ISGRC Grade A. The leaks experienced by the GI group trended towards being more likely to be Grade C. Without proximal diversion, a column of solid stool can form and reach the level of the anastomotic leak, potentially worsening the mechanical defect as well as resulting in the extra-luminal spillage of stool.^57,58^ This is in keeping with previous literature, demonstrating that LIs do not necessarily reduce the incidence of anastomotic leak, but rather can significantly decrease the risk of uncontrolled pelvic sepsis requiring urgent re-operation.^59,60^ Nonetheless, amongst the included studies, only 2 patients in the GI group required formation of a permanent end colostomy.

The strengths of the present systematic review and meta-analysis include the number of included studies and patients, the comprehensive risk of bias and GRADE assessments, and novelty. The study limitations include the low to very-low certainty of evidence, inclusion of mostly observational data, and heterogeneity. Given the nature and consequences of the intervention, it is likely that the observational data were influenced by selection bias. In the comparative studies included in the present meta-analysis, patients in the GI and LI groups were fairly well matched in terms of demographics, comorbidities, operative approaches, disease processes, and likelihood of receiving neoadjuvant radiotherapy. Nonetheless, the risk of residual confounding in the included studies is high given the lack of propensity score analyses. Significant heterogeneity was present between studies in outcome reporting, which limited the statistical power of the meta-analyses. Specifically with regards to cost analyses, only a single study compared total inpatient costs of GI and LI, demonstrating a significant reduction in cost with the use of GI.^19^ Without more uniform outcome reporting, however, we were unable to comment on the relative costs of these techniques. Operative techniques also demonstrated significant between-study heterogeneity. Specifically, techniques for GI creation varied widely. Some studies reported parietal splitting techniques, while others did not. The material used to oppose the loop of ileum to the anterior abdominal wall varied (eg, vessel loops, suture, etc.) and the techniques for fixing these materials at the level of the skin were also variable (eg, suture, gauze). This heterogeneity could have significantly impacted outcomes such as postoperative morbidity and LOS. The GRADE assessment of the overall quality of evidence was significantly impacted by indirectness and imprecision, and thus larger prospective studies with standardized inclusion criteria, exclusion criteria, and outcome measures would benefit the current body of literature. Currently, given the low to very-low certainty of evidence, conclusions regarding the specific clinical instances in which GI may be of benefit remain difficult to deduce.

Overall, the use of GIs in patients undergoing TME for rectal cancer with reconstruction of gastrointestinal continuity via a low-to-medium risk colorectal anastomosis does not appear to increase the risk of anastomotic leak or postoperative morbidity compared to the use of LIs. In select patients, this may be a feasible operative technique that avoids the need for an LI, along with its potential stoma-related morbidity and adverse effects on QoL. These data offer low to very-low certainty evidence and thus further large prospective comparative studies are warranted to evaluate the use of GI in patients deemed to be at low-to-medium risk of anastomotic leak.

## Supplemental Material

Supplemental Material - Ghost Ileostomy Versus Loop Ileostomy Following Oncologic Resection for Rectal Cancer: A Systematic Review and Meta-Analysis Click here for additional data file. Supplemental Material for Ghost Ileostomy Versus Loop Ileostomy Following Oncologic Resection for Rectal Cancer: A Systematic Review and Meta-Analysis by Tyler McKechnie, MD, Jay Lee, MD, Yung Lee, MD, Léa Tessier, BSc, Nalin Amin, MD, Aristithes Doumouras, MD, MPH, Dennis Hong, MD, MSs, and Cagla Eskicioglu, MD, MSc in Surgical Innovation
