# Supplementary material for: Ghost Ileostomy Versus Loop Ileostomy Following Oncologic Resection for Rectal Cancer: A Systematic Review and Meta-Analysis
Source: Surg Innov. 2023 Apr 4;30(4):501–16. doi: 10.1177/15533506231169066 (PMC10403968; doi:10.1177/15533506231169066)
Supplement: Supplemental Material - Ghost Ileostomy Versus Loop Ileostomy Following Oncologic Resection for Rectal Cancer: A Systematic Review and Meta-Analysis [file sj-pdf-1-sri-10.1177_15533506231169066.pdf]

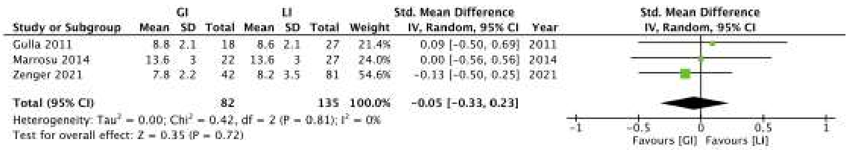

Length of Stay – Random effect inverse variance meta-analysis comparing ghost ileostomy and loop ileostomy

209x279mm (72 x 72 DPI)

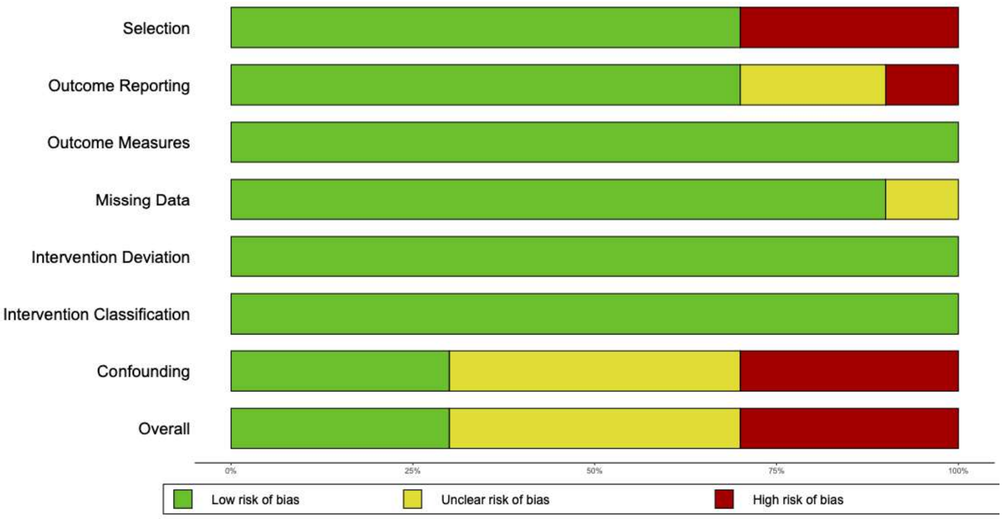

Supplemental Figure 2. Pooled risk of bias analysis according to the Risk of Bias in Non-randomized Studies of Interventions (ROBINS-I) for included observational studies.

387x206mm (72 x 72 DPI)
